# Conditional Approval Pathways: The “Special” Case of Global Regenerative Medicine Regulation

**DOI:** 10.1089/rej.2017.1932

**Published:** 2017-02-01

**Authors:** James A. Smith, David A. Brindley

**Affiliations:** ^1^ Nuffield Department of Orthopaedics, Rheumatology and Musculoskeletal Sciences, Nuffield Orthopaedic Centre, University of Oxford, Oxford, United Kingdom.; ^2^ The Oxford–UCL Centre for the Advancement of Sustainable Medical Innovation (CASMI), University of Oxford, Oxford, United Kingdom.; ^3^ Harvard Stem Cell Institute, Harvard University, Cambridge, Massachusetts.; ^4^ Department of Paediatrics, University of Oxford, Oxford, United Kingdom.; ^5^ Centre for Behavioural Medicine, UCL School of Pharmacy, University College London, London, United Kingdom.; ^6^ Stanford-UCSF FDA Centre for Regulatory Science and Innovation, San Francisco, California.

Biomedical innovation and the societal benefits it catalyses is essential and challenging. Yet from reductionist viewpoint, the term *innovation* has been consistently misapplied to describe the process of translating scientific advancements from the laboratory to patient bedside. It is easy to forget that etymologically, *innovation* stems from Latin roots meaning to *change or develop* and *introduce*.

Historically however, and perhaps detrimentally, a disproportionate quantum of resource has been directed toward research into fundamental laboratory science (*changes or developments*)—neglecting the regulatory frameworks guiding therapeutic development and clinical adoption (*introduction*). This has also lead to a discontinuity in efforts to better evaluate and rationalize regulatory frameworks, that are often dismissed as “someone else's problem.” (Or as Douglas Adams would coin, “an SEP field.”)

The central thesis discussed herein is that the regulatory incentives presently afforded to developers of regenerative medicines—spanning the full ambit of cell therapy, gene therapy, and tissue engineering—may not be instantaneously proportional or rational. That is to say, when such accelerated pathways were designed and instituted, this was based on different levels of understanding—in terms of regenerative medicine technology platforms and accelerated regulatory approaches—than we have today, based on empirical experience. And as such, may not be optimal to support the long-term sustainability of the regenerative medicine industry nor maximize the transferability of regulatory advances in regenerative medicine to support other therapeutic platforms.

We support regenerative medicine innovation and believe that regulators and basic scientists deserve substantial credit and gratitude for the advances made, accelerating novel therapeutics and devices to patients with unmet medical needs. The focus of our appraisal is simply:

1. What have we learnt from accelerated regulatory pathways in regenerative medicine?
2. As the regenerative medicine industry and underpinning technologies mature, how regulatory pathways be continuously developed to ensure their rationality and proportionality?
3. Is it feasible to apply the accelerated regulatory pathways “piloted” in regenerative medicine to other emerging technological platforms?

Regulatory innovation must be encouraged and aligned with scientific innovation. However, as a field, it is critical that we pause to evaluate the lessons learnt to date, to ensure consistent momentum and patient benefit in the future.

In certain circumstances of considerable unmet clinical need, departures from conventional approval processes can be justified to accelerate access to those potentially life-saving medicines. As such, novel regulatory pathways have been implemented by many of the major regulatory bodies, including the U.S. Food and Drug Administration (FDA), the European Medicines Agency (EMA), and Japan's Pharmaceuticals and Medical Devices Agency (PMDA). The FDA, for example, has developed four approaches to improve the speed of approval processes: priority review, breakthrough therapy, accelerated approval, and fast track,^1^ and EMA has introduced accelerated assessment and adaptive pathways. Both PMDA and EMA have introduced conditional approval pathways.^2,3^

Under conditional approval pathways, such as conditional marketing authorization in EMA, medicinal products with promising, but not comprehensive, efficacy data are given market authorization on the condition that the product is further evaluated while on the market. After a given period, therapies may be withdrawn from the market, granted “standard” approval, or continue to be marketed conditionally, depending on the data generated during that time. The scope of conditional approval pathways varies marginally by jurisdiction, but they are typically reserved for medicinal products addressing life-threatening or seriously debilitating conditions. Under the EMA conditional approval pathway, for example, the scope encompasses products for seriously debilitating diseases or life-threatening diseases, those for use in emergency situations, and orphan medicinal products.^4^ A number of therapies have been approved under these pathways: between 2007 and 2015, 17 therapies received EMA conditional approvals, while 25 received FDA accelerated approvals.^5^ In both jurisdictions, most of these approvals were in the oncology area and most were designated as orphan drugs, thus providing therapeutics to patients with potentially very limited alternative choices.

Conditional approval pathways have not gone without controversy. Although they aim to accelerate patient access to medicines, some analyses have in fact shown that development time from first-in-human testing to marketing authorization is comparable to full marketing authorization, yet are based on less comprehensive data.^6^ That led the authors to question if companies, instead of using the pathway prospectively, are using it as a backup to full authorization when data packages may not be strong enough to support a full market authorization through conventional pathways. Early access pathways in the United States have attracted attention recently, as Sarepta Therapeutics' drug Exondys (eteplirsen) gained accelerated approval based on clinical data from just 12 patients.^7,8^ It is possible that in such scenarios, potential limitations in clinical data sets have led to parallel pseudo-evaluation of nonclinical factors.

For example, social media reports and excerpts from investor presentations suggest that there was a utilitarian, as well as scientific, basis to the FDA evaluation of Sarepta. “Dr Woodcock [Director of FDA CBER] cautioned that if Sarepta did not receive accelerated approval for eteplirsen, it would have insufficient funding to continue to study eteplirsen and the other similar drugs in its pipeline.”^9,10^ Thus, a move away from evaluation purely of the risks and benefits of the specific drug in question, to commercial considerations (which could also be considered utilitarian), may be taking place, where historically a “Church and State” relationship between commercial and noncommercial factors in evaluating therapeutic candidates has been encouraged. Interestingly, although some would argue that this approval could result in patients paying for ineffective therapeutics, there was exceptionally strong support for the approval among patient groups. We do not comment on the appropriateness of this decision; instead, we emphasize that accelerated pathways be consistently applied in a manner that ultimately provides the greatest benefit possible to patients, whether this is directly or via a less traditional, more utilitarian approach.

In Japan, substantial regulatory incentives have been directed toward regenerative medicine, arguably in part driven by political factors and substantial national investments in induced pluripotent stem cell (iPSC) technology platforms. In 2014, Japan introduced a conditional approval system in which regenerative medicine products could be approved based on data which demonstrate safety and are “likely to predict efficacy.”^3^ Unlike other pathways recognized by the International Conference on Harmonisation of Technical Requirements for Registration of Pharmaceuticals for Human Use (ICH) countries, of which Japan is one, this regulatory approach prioritizes one therapeutic approach over others, rather than focusing purely on the medical need of the cohort at which the therapy is aimed.

The efficacy of conditionally approved therapeutics is often not fully understood, and, therefore, payers, including patients, in part fund the continuing evaluation of the therapy after approval. If commercial considerations were included in approval decisions, given that regenerative medicines are typically very costly, one could go so far as to argue that *greater* efficacy data should be required for regenerative medicines when compared to other, cheaper therapeutic approaches before approval! Undoubtedly, the existence of this system has catalyzed investment in regenerative medicines in Japan; the question is: has this come at the expense of investment in other technologies that might have generated a greater return to patients?

A recent proposed change to U.S. Law, the REGROW Act, appeared to closely reflect the premise on which the Japanese system is based. Announced on March 16, 2016, the proposed amendment to the Federal Food, Drug and Cosmetic Act outlined an approval process for cellular therapies, separate to that of other therapeutics, in which products are granted conditional market authorization without initiation of phase III trials, based on “preliminary evidence of safety, and a reasonable expectation of effectiveness.”^11^ This act was ultimately not enacted, but its appearance may reflect a growing trend for prioritization of particular technologies rather than particular patient needs. Similarly, in Korea, in 2012, a proposal for approval of stem cell therapies based purely on Phase I or investigator-led studies was proposed.^3^ Perhaps particular technologies and particular patient needs are intrinsically linked; perhaps they are not. Data-driven evaluation of the existing pathways will enable determination of the most effective regulatory pathways.

Returning to our original questions, what can we determine?

1. What have we learnt from accelerated regulatory pathways in regenerative medicine?

Most importantly, we have learnt that accelerated access programs are not only of conceptual interest but also they can actually deliver earlier and expanded patient access to innovative therapeutics. Additionally, it is evident that novel regulatory vehicles can act to mobilize healthcare stakeholders—including patient groups and investors—almost as much as the underlying therapeutics. All changes, however, create uncertainty, opportunity, and (short-term) potential conflict.

In regenerative medicine, short-term conflicts have emerged by virtue of the closer interaction between the operations and viewpoints of product developers, regulators, and payers, all of whom have operated in an arms-length manner historically. This additional transparency is likely to confer increased productivity and lower risks in the biomedical innovation ecosystem as a whole in time. Yet, in the short term, it is akin to a constitutional change in the evolution of a state, from one controlled by a single ecclesiastical body or monarchy, to a more decentralized federal state. It takes time for a clear national identity and operational systems to emerge, with opportunities for sustained multistakeholder debates and lobbying in the interim.

2. As the regenerative medicine industry and underpinning technologies mature, how can regulatory pathways be continuously developed to ensure their rationality and proportionality?

Central to this issue is the extent and rate at which the inherent technological risks and uncertainties of regenerative medicine as a technological platform decrease with the passage of time, during which research and development and clinical experience accumulate. Therefore, the extent to which the regulatory challenges faced by regenerative medicine cease being extraordinary and unsolved, versus ordinary and effectively addressed by the prevailing regulatory system of the day, is central to determine the rationality and proportionality of accelerated approvals in the long term to support regenerative medicines.

If adaptive pathways remain the status quo for all regenerative medicine clinical studies, even as the industry reaches maturity, the logical conclusion is that adaptive pathways are central to all regulatory vehicles of the future, replacing established linear and sequential approaches. Conversely, if such adaptive instruments are only used to “kick start” an industry, then are regenerative medicine product developers of tomorrow to be forced to pursue slower and higher risk regulatory pathways? If so, regenerative medicine may become a less attractive area for commercial investment and academic investigation.

Therefore, future evaluations of this question must consider the rationality and proportionality of novel regulatory vehicles in the dimensions of both short-term innovation risk (a proxy for technological novelty and thus inherent uncertainty) and longitudinal risk (a proxy for time: technology development time and time in which the novel regulatory vehicles and multistakeholder understanding increases). In some respects, this concept has similar conceptual origins to option pricing models in finance.

3. Is it feasible to apply the accelerated regulatory pathways “piloted” in regenerative medicine to other emerging technological platforms?

The infancy of the regenerative medicine regulatory pathway in Japan renders this question challenging to answer. Perhaps, the best method to answer it will lie in analysis of the existing broader accelerated pathways. Examining these pathways might allow determination of success across different technological platforms; if the model has proven particularly effective for regenerative medicines, other emerging platforms could similarly benefit.

***

Today, regenerative medicine and the regulatory framework in which it operates is in a period of transition: from innovative start-up curiosity to established pillar of the biotech industry. There is an unavoidable lag between the maturity of the underpinning science and corresponding regulatory framework, which develops at a later date in response to need. Crudely, this is akin to staying in a hotel for a few weeks in between apartments.

*In a Room with a View*—which opens with a description of the protagonists exchanging rooms in a Florencian hotel—E.M. Forster divides physical restrictions into two types: “It makes a difference, doesn't it, whether we fence ourselves in, or whether we are fenced out by the barriers of others?” In regenerative medicine, multistakeholder support—particularly from regulators—has allowed us to determine and adapt our own restrictions.

Our efforts must now be focused on ensuring the success of these efforts as we mature as an industry [toward our permanent lodgings—at the forefront of biotech] and ensure that we do all we can to ensure the transferability of the regulatory innovations from which we have benefited to the developers of other emerging therapeutic platforms.

We now need to share our “Room with a View” with other innovators.
